# Nonadditive effects of two contrasting introduced herbivores on the reproduction of a pollination‐specialized palm

**DOI:** 10.1002/ecy.3797

**Published:** 2022-07-27

**Authors:** Raquel Muñoz‐Gallego, Jose M. Fedriani, Pau E. Serra, Anna Traveset

**Affiliations:** ^1^ Global Change Research Group, Mediterranean Institute of Advanced Studies (IMEDEA, CSIC‐UIB) Esporles Spain; ^2^ Desertification Research Centre (CIDE, CSIC) Moncada Spain; ^3^ Doñana Biological Station (EBD, CSIC) Seville Spain

**Keywords:** *Chamaerops humilis*, goat, herbivory, inflorescence damage, introduced herbivore, leaf damage, nonadditive effects, palm, *Paysandisia archon*, pollination‐specialized plant, reproductive success

## Abstract

Plant–animal interactions fall within a mutualism–antagonism continuum, exerting a wide range of effects on plant reproductive success. These effects become even more complex and diverse when several disparate animal species interact with the same plant species. Despite the increasing number of studies about the influence of herbivory on plant performance, the outcomes mediated by pollination and the combined impact of multiple herbivores on pollination‐specialized plants are underexplored. In this study, we chose the Mediterranean dwarf palm *Chamaerops humilis* (Arecaceae) to illustrate the isolated and joint effect of two contrasting introduced herbivores, the palm borer *Paysandisia archon* (Lepidoptera, Castniidae) and feral goats, on pollinator abundance and plant reproductive success. To this aim, we monitored moth herbivory and goat herbivory in four palm populations in Mallorca (Balearic Islands) during 2019 and 2020. The effect of herbivory varied widely depending on both the herbivore and the pollinator species. Moth herbivory had a positive effect on pollinator abundance and fruit initiation, whereas goat herbivory had a negative effect on inflorescence production, pollinator abundance and fruit initiation. In addition, both herbivores exerted unexpected nonadditive effects on palm reproduction. Palms attacked by both herbivore species produced many more inflorescences (up to 18‐fold) but had a lower fruit initiation success (close to zero) than unattacked palms or those attacked by a single herbivore species. Interestingly, only one of the two main pollinator species (the nitidulid beetle *Meligethinus pallidulus*) was impacted by herbivory. Our study highlights the need to investigate the possible nonadditive effects of all coexisting herbivores on plant performance, especially when establishing conservation plans and pest control strategies.

## INTRODUCTION

Herbivory is a plant–animal interaction typically classified as an antagonism due to its numerous direct and indirect negative effects on plant pollination and reproductive success (Barber et al., 2012; Bronstein et al., 2007; Haas & Lortie, 2020; Lucas‐Barbosa, 2016; Moreira et al., 2019). However, all interspecific ecological interactions fall along a mutualism–antagonism continuum (Bronstein, 2015; Perea et al., 2013) and influence each other within complex multilayered networks (Pilosof et al., 2017; Strauss & Irwin, 2004). Thus, the impact of herbivory on plant performance can also be neutral or even positive. Plants sometimes activate induced responses to damage that boost reproduction, i.e., they overcompensate (Garcia & Eubanks, 2019; Owen & Wiegert, 1976), for instance by increasing seed/fruit production (Aguirrebengoa et al., 2021), or promoting pollinator attraction (Cozzolino et al., 2015).

The effects of herbivory become more complex and diverse when the plant is attacked by several disparate herbivorous species (Stephens et al., 2013) because (1) they can differ in their life cycle, body size, and behavior, exerting contrasting damage rates on different plant tissues. For instance, large mammals usually consume great amounts of assorted plant tissues in a very short time, while insects cause more localized and long‐lasting damage (Gómez & Zamora, 2000; Kotanen & Rosenthal, 2000; Perkovich, 2021); (2) plants can activate disparate mechanisms of defense and respond specifically to each herbivore species (Rusman et al., 2018); and (3) herbivores can directly interact among them, modulating the sign and strength of their net effects on plant fitness (Lecomte et al., 2017; Ohgushi, 2005). Thus, the joint effect of two different herbivores could be equal to the effect of one of them (i.e., a dominance effect) or be the sum of the individual effects (i.e., an additive effect). However, when herbivores interact, the effect of one herbivore can change with the intensity of herbivory by the second species, resulting in unexpected nonadditive effects on plant reproductive success (Aguirrebengoa et al., 2021; Côté et al., 2016; Gagic et al., 2016). Most studies on the joint impact of different herbivores on plant performance focus on phytophagous insects but few on disparate herbivore species such as invertebrates and vertebrates (but see Anderson & Paige, 2003; Gómez & Zamora, 2000; Perkovich, 2021).

Given that introduced herbivores represent one of the major threats to native flora (Nuñez et al., 2010; Pisanu et al., 2012), assessing their effects on plant communities and, especially, on singular plant species, could be crucial for biodiversity maintenance. In this context, pollination‐specialized plants, which only rely on one or a few pollinator species, might be particularly vulnerable to herbivory (Glaum & Kessler, 2017; Lybbert & St. Clair, 2017), as it has been described for other types of disturbance (Bennett et al., 2020; Memmott et al., 2007; Rodger et al., 2021). For instance, the reproduction of the pollination‐specialized *Yucca baccata* was critically impacted by floral herbivory since pollinator visitation and fruit production were null for herbivore‐attacked plants (Lybbert & St. Clair, 2017). Thus, pollination‐specialized plants are probably less likely to compensate for pollinator failure with alternative pollinators (Wilcock & Neiland, 2002).

On island ecosystems, the impact of herbivory is especially severe as their food webs are simpler, and the endemicity rate is usually very high (Bellard et al., 2017; Traveset & Richardson, 2014). Feral goats, in particular, are among the most destructive alien mammals on islands, having devastating effects on native flora (Capó et al., 2021; Chynoweth et al., 2013; Gizicki et al., 2018). Specifically, on Mallorca island (Balearic Islands, Spain), the abandonment of agriculture in the 1960s contributed to a progressive expansion of feral goat (*Capra hircus* L.) populations throughout the mountains, currently reaching more than 20,000 individuals (Vives & Baraza, 2010). In addition, a more recently introduced herbivorous species, the neotropical palm borer *Paysandisia archon* Burmeister (Lepidoptera, Castniidae), is severely damaging the natural populations of the dwarf palm *Chamaerops humilis* L. (Arecaceae), the only native palm in the western Mediterranean region (Guzmán et al., 2017). Despite the existing knowledge on the basic ecology of both introduced herbivores on the island (Capó et al., 2021; Rivera Sánchez, 2014; Ruiz et al., 2017; Sarto i Monteys & Aguilar, 2005), no research has yet been conducted neither on their respective isolated effects nor on their joint impact on plant reproductive success.

In this study, we selected the pollination‐specialized palm *C. humilis* in Mallorca to illustrate how the isolated and combined effects of two contrasting introduced herbivores, the invasive moth *P. archon* and feral goats, impact pollinator abundance, and, ultimately, palm reproductive success. First, we hypothesize that palms attacked by *P. archon* and/or goats will produce fewer inflorescences than unattacked palms due to the reallocation of resources in response to herbivory. Second, we predict herbivory will negatively influence pollinators' visits, resulting in a lower fruit set. Third, these two contrasting herbivores might exert nonadditive effects on pollinator abundance and palm reproductive success (Côté et al., 2016). As herbivory effects on plant‐pollinator interactions could depend on the pollinator's behavior and biology (Bruinsma et al., 2014; Jacobsen & Raguso, 2018), we also tested the predictions mentioned above for each of the two main palm pollinator species (the weevil *Derelomus chamaeropis* and the pollen beetle *Meligethinus pallidulus*) separately.

## METHODS

### Study system

**FIGURE 1:**
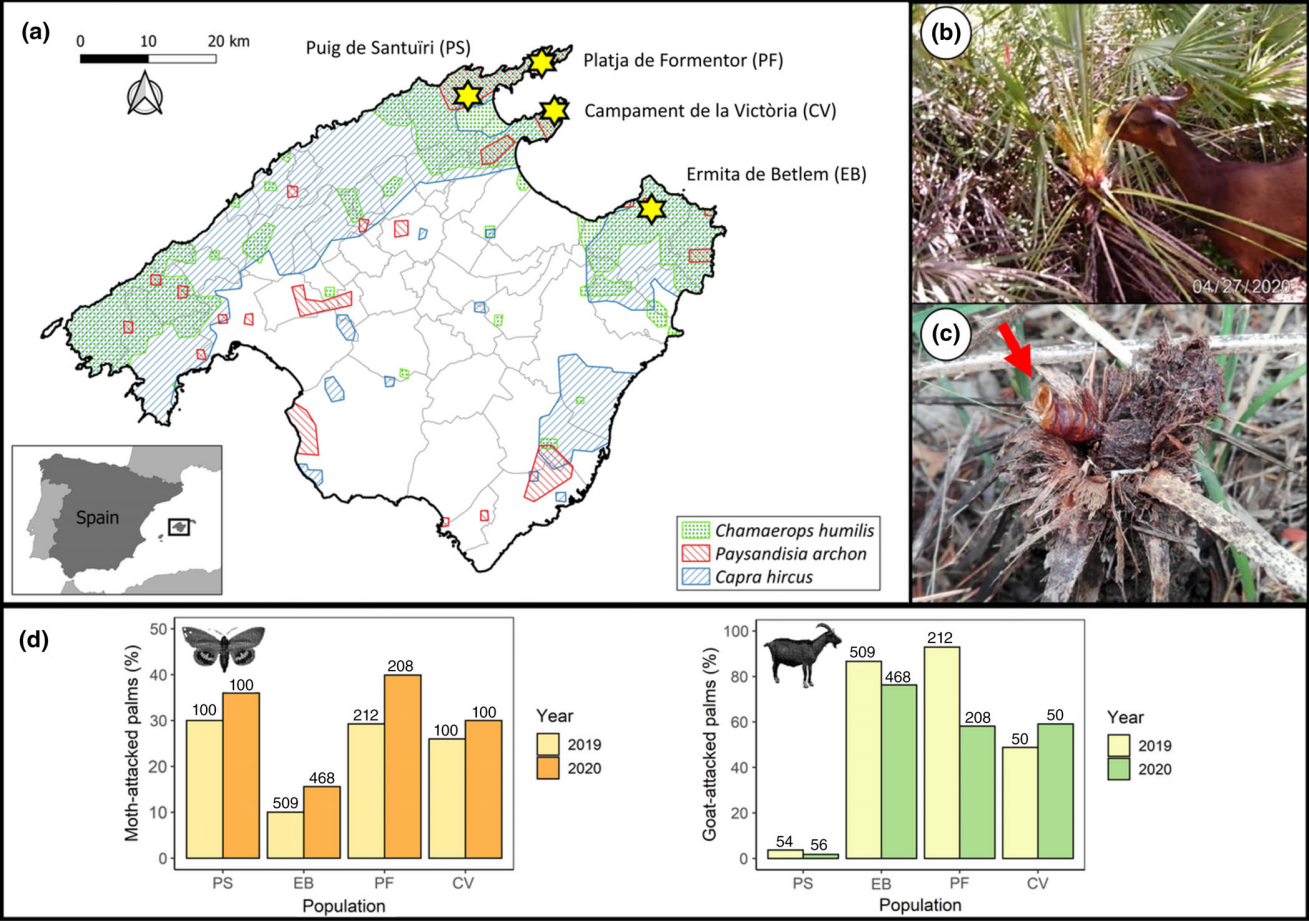
(a) The four study areas (yellow stars) selected on the island of Mallorca (Balearic Islands, Spain) and the distribution of *Chamaerops humilis* (green), *Paysandisia archon* (red) and *Capra hircus* (blue; modified from CAIB, 2021). (b) Feral goat eating *C. humilis* inflorescences. (c) A pupal exuviae (red arrow) and sawdust produced by *P. archon* larvae emerging from a dead stem of *C. humilis*. (d) Proportion of moth‐attacked palms (left) and goat‐attacked palms (right) for each population and sampling year. Sample size, i.e., number of palm individuals, is indicated on each bar.

The dwarf palm *C. humilis* is distributed throughout the western Mediterranean region (Guzmán et al., 2017), being relatively abundant in Mallorca, especially in the Serra de Tramuntana and the Serra de Llevant (see Figure 1a). This dioecious plant blooms in March–April, fruits start to develop in May–June, and fruit ripening takes place in September–November. Flowers form dense inflorescences that develop within a single enlarged bract. Fruits are polydrupes, usually comprising one to three drupes (Herrera, 1989). In our study, we will apply the term “fruit” to each drupe, as it behaves as an independent pollination and dispersal unit. At anthesis, both male and female plants emit volatile compounds, mostly from the leaves (Dufaÿ et al., 2003), that mainly attract two pollinator species, the specialist weevil *D. chamaeropis* (Curculionidae; Anstett, 1999) and the pollen beetle, *M. pallidulus* (Nitidulidae; García et al., 2018). In the nursery pollination system formed by *C. humilis* and *D. chamaeropis*, weevil larvae develop from eggs laid the previous spring inside the rachis of persistent old inflorescences, more frequently and successfully within male than within female inflorescences (Jácome‐Flores et al., 2018). Although it has been identified as a floral visitor of other plant species (see Cursach & Rita, 2012), *M. pallidulus* also seems to be strictly associated with male inflorescences of palms (Audisio et al., 2009; Jelínek et al., 2011) since its larval development takes place on the inner surface of dwarf palm bracts (Ponel & Lemaire, 2012; R. Muñoz‐Gallego, personal observation). Other visitors were found within the inflorescences although very infrequently, thus we do not consider them as potential effective pollinators (see Appendix S1: Table S1). To date, very few studies have reported herbivory on the dwarf palm, and all of them focus on vegetative tissues (e.g., Hanane & Amhaouch, 2021; Rivera Sánchez, 2014; Salinas & Guirado, 2002). In Mallorca, specifically, there is good evidence that goats not only act as seed dispersers of this palm (Muñoz‐Gallego et al., 2019) but also as herbivores, browsing on leaves (Rivera Sánchez, 2014) and inflorescences (R. Muñoz‐Gallego, personal observation; Figure 1b). Among the invertebrate herbivores, the alien invasive moth *P. archon* is spreading all over the island and its attack on the dwarf palm is getting worrisome (Ruiz et al., 2017). This neotropical moth was first reported in Mallorca in 2003 (Sarto i Monteys & Aguilar, 2005) and it has been recorded in more than 10 European countries so far, becoming a serious pest of palm trees (Muñoz‐Adalia & Colinas, 2020). Adult flight and egg laying happen from mid‐May to late September. Eggs are deposited on the crown of the palm, and larvae feed inside the stem and the young leaves for 1 or 2 years (annual or biannual cycle), eventually causing the palm's death (Figure 1c; Sarto i Monteys & Aguilar, 2005).

### Study areas

The study was carried out on the island of Mallorca (Balearic Islands, Spain) during 2019 and 2020. Specifically, four dwarf palm populations were chosen (Figure 1a and Appendix S1: Figure S1): Ermita de Betlem (EB) (39°44′20.5″ N 3°18′52.4″ E), Puig de Santuïri (PS) (39°52′42.6″ N 3°01′56.9″ E), Platja de Formentor (PF) (39°55′54.9″ N 3°08′11.4″ E), and Campament de la Victòria (CV) (39°52′10.0″ N 3°09′36.9″ E). All study areas consist of coastal shrublands dominated by Mediterranean species such as *Cistus monspeliensis*, *Ampelodesmos mauritanicus*, *Asphodelus aestivus*, *Smilax aspera*, *Pistacia lentiscus*, *Olea europaea* var. *sylvestris*, *Pinus halepensis*, and *Quercus ilex*. The climate of the Balearic archipelago is typically Mediterranean, characterized by two rainy seasons, a hot dry summer, and a mild winter. The most rain falling was in October 2019 and April 2020, and there was an extreme drought in June 2019. Annual average temperature was ~18.5°C during both study years (data from Red de Estaciones Meteorológicas de Baleares).

### Effects of moth herbivory on pollinator abundance and palm reproductive success

To study the effects of moth larvae feeding (hereafter, moth herbivory) on palm reproduction, we selected 30 palm females and 30 males, half unattacked and half attacked of each sex whenever possible, in the four palm populations. We considered as moth‐attacked palms those with any visible sign of infection: presence of sawdust in the stems, perforated leaves, dried core leaves, twisted or dead stems (Sarto i Monteys & Aguilar, 2005). Overall, pooling the four populations, we sampled 98 females (176 inflorescences) and 88 males (164 inflorescences) in 2019, and 109 females (194 inflorescences) and 77 males (138 inflorescences) in 2020. Since some individuals from 2019 did not bloom again in 2020, they were replaced by new ones to increase our sample sizes. The sampling was conducted between April and May, at the peak of the flowering period. We registered palm size (i.e., the total number of stems) and inflorescence production (i.e., the total number of inflorescences produced) for each palm in both sampling years. In one to three intact inflorescences at anthesis per palm, the abundance of *D. chamaeropis* and *M. pallidulus* individuals per inflorescence was recorded during one 3‐minute census (García et al., 2018). These censuses were conducted between 09:30 and 17:00 (solar time) on sunny and windless days (12 and 16 days in 2019 and 2020, respectively). For female inflorescences, we also registered the initial number of flowers to estimate later the fruit set. We calculated the pollination success via sequential components following Fedriani et al. (2015): fruit initiation (i.e., [flowers that initiate fruit]/[initial number of flowers]) and fruit development (i.e., [number of developed fruits]/[number of initiated fruits]). Flowers that initiated fruit were identified as those with brown stigmas (i.e., fertilized flowers) in June, whereas the number of developed fruits was recorded in September. It is important to highlight that about half of the female inflorescences selected to estimate fruit set were partially or fully consumed by goats between June and September. Thus, most of them did not initiate fruits (see Appendix S1: Figure S2). These inflorescences were excluded to analyze the isolated effects of moth herbivory (see also *Statistical analyses*).

### Effects of goat herbivory on pollinator abundance and palm reproductive success

To evaluate the effects of goat herbivory, we differentiated between herbivory on leaves (hereafter, leaf damage) and herbivory on inflorescences (hereafter, inflorescence damage) since they could lead to different effects on plant reproduction (McCall & Irwin, 2006). At the beginning of both sampling seasons (late March), we registered leaf damage (i.e., the proportion of browsed leaves, estimated in 15 randomly selected leaves) and inflorescence damage (i.e., the proportion of inflorescences eaten, partially or fully, over the total number of inflorescences produced) in the same individuals used to estimate the effect of moth herbivory. We excluded the PS population due to the extremely low incidence of goat herbivory (see Figure 1d). Overall, we sampled 72 females (126 inflorescences) and 60 males (107 inflorescences) in 2019, and 79 females (138 inflorescences) and 51 males (88 inflorescences) in 2020. Because we did not find any correlation between inflorescence damage and leaf damage (Spearman's *r*_s_ = −0.03, *p* = 0.58), we considered both variables separately. Inflorescence production, pollinator abundance, fruit initiation, and fruit development were measured as described in *Effects of moth herbivory on pollinator abundance and palm reproductive success*. However, in this case, flower losses due to goat inflorescence damage (mostly taking place during March) were accounted for in our estimates of fruit initiation. Finally, to verify the consumption of *C. humilis* inflorescences by goats and other potential mammal herbivores (e.g., *Rattus rattus* and *Oryctolagus cuniculus*), we registered herbivore visits and inflorescence damage events (considering only those visits with inflorescence consumption) by camera trapping (Figure 1b). Overall, we recorded 32 flowering palms (15 females and 17 males) in the four study sites (*n* = 7 for EB, *n* = 7 for PS, *n* = 8 for PF, and *n* = 10 for CV) for 14 consecutive days on average (336.75 ± 21.46 h per palm; mean ± SE) in spring 2020. For further details, see Appendix S1.

### Statistical analyses

To assess the isolated and combined effects of both herbivores on palm reproduction, we fitted three different generalized linear mixed models (GLMMs; Zuur et al., 2009): (1) “Moth herbivory models” to test the isolated effects of moth herbivory; the predictor variable was whether the palm was attacked or not by *P. archon* (*Mh*). The goat‐attacked palms, regardless of the type of damage, were excluded from the analyses. (2) “Goat herbivory models” to determine the isolated effects of goat herbivory; here, the predictor variables were inflorescence damage (Infdam), leaf damage (Leadam), and their second‐order interaction. In this case, the moth‐attacked palms were excluded from the analyses. (3) “Interaction models” to investigate potential nonadditive effects by both herbivores; the predictor variables were Mh, Infdam, Leadam, as well as their second‐ and third‐order interactions. We used all sampled individuals from the populations where palms were distinctly affected by both herbivores (i.e., EB, CV, and PF). When interactions among types of herbivory were not statistically significant, they were removed from the models and the significance of all remaining main and interaction effects re‐checked. These three types of models were run for each response variable: inflorescence production, number of *D. chamaeropis* individuals per inflorescence, number of *M. pallidulus* individuals per inflorescence, overall pollinator number per inflorescence (i.e., the sum of numbers of both pollinator species), fruit initiation, and fruit development. The first four response variables were fitted with a negative binomial error distribution and a log link function. The last two variables were fitted with a quasi‐binomial error distribution and a logit link function (to account for overdispersion). Palm sex (*S*) and the total number of available inflorescences per palm (Ti, i.e., excluding those consumed by goats) were included as covariates in most models, as their effect on pollinator abundance and fruit set has been proved in previous studies (e.g., García et al., 2018). Palm size (Ps) was also included as a covariate in the models for inflorescence production. However, it was not for the other response variables due to its strong and positive correlation with Ti (Spearman's *r*_s_ = 0.44, *p <* 0.001). These covariates were removed from the models when they had no effect on the response variable. Individual palm nested within the palm population was specified as a random effect to control for the variation among palm individuals and populations. Finally, to control for annual variation, we added the sampling year (*Y*) as a fixed effect and its interaction with every fixed term. We fitted all GLMMs using the R functions *glmmTMB* (glmmTMB) and *glmmPQL* (MASS). For each factor level, we calculated the adjusted means and standard errors using the *lsmeans* (lsmeans) function in R software (R Core Team, 2022).

## RESULTS

### The isolated effects of moth herbivory on palm reproduction

**FIGURE 2:**
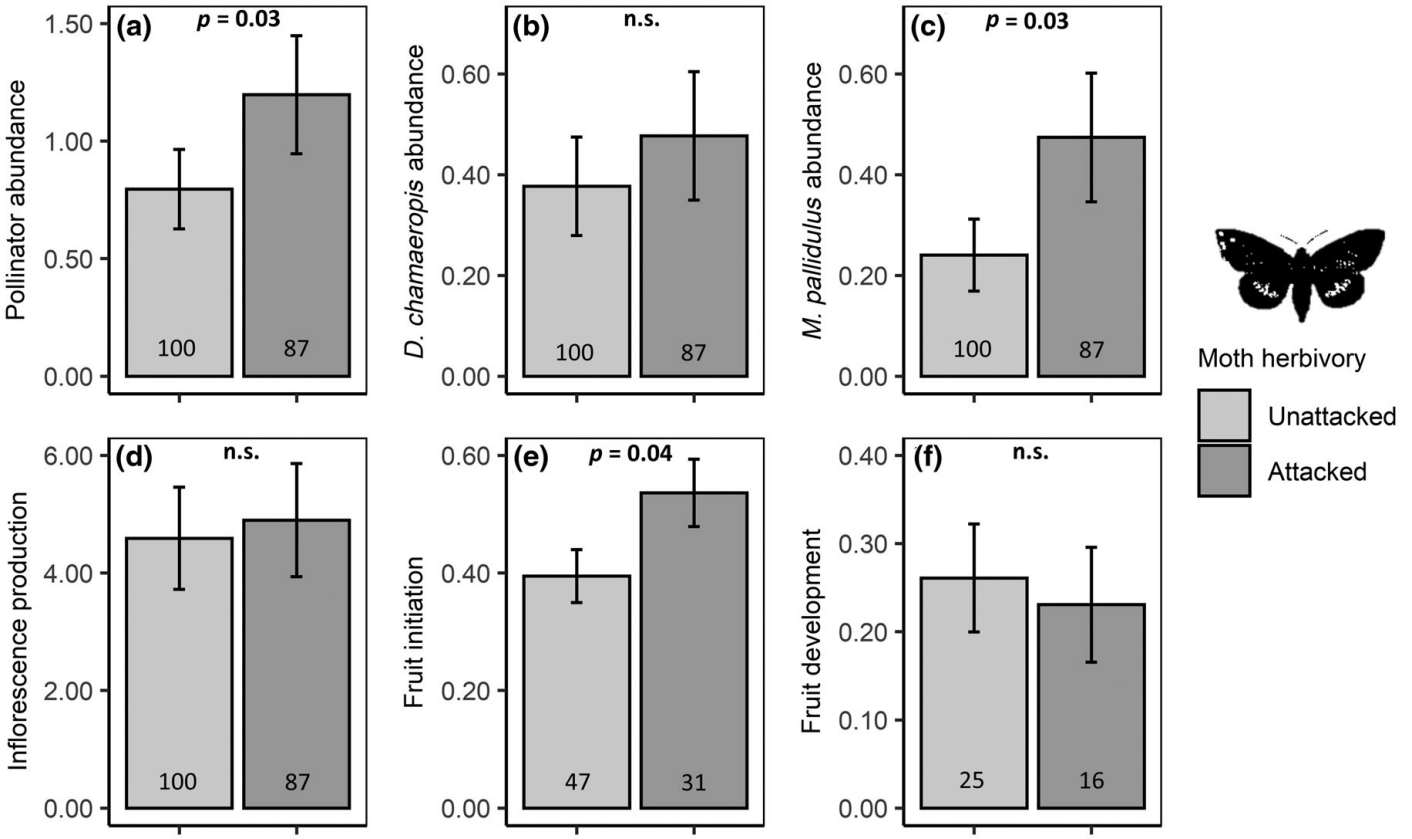
Isolated effects of moth herbivory on palm reproduction. Model‐adjusted means (± standard error) of (a) overall pollinator abundance per inflorescence, (b) *Derelomus chamaeropis* abundance per inflorescence, (c) *Meligethinus pallidulus* abundance per inflorescence, (d) inflorescence production, (e) fruit initiation, and (f) fruit development, depending whether the palm is moth‐attacked or not. *N* is indicated within each bar as the number of palm individuals. For fruit development, we only used data from 2020 due to insufficient *N* in 2019. The *p* values denote statistically significant effects and n.s. indicates nonsignificant effects.

The proportion of palms attacked by *P. archon* varied among populations, ranging from 10.0% (EB, 2019) to 39.9% (PF, 2020), being consistently higher in the second sampling year (see Figure 1d). Moth herbivory had no effect on inflorescence production (χ^2^ = 0.33, df = 1, *p* = 0.57; Figure 2d). However, moth‐attacked palms had a 1.5‐fold higher overall pollinator abundance per inflorescence than unattacked ones (χ^2^ = 4.50, df = 1, *p* < 0.05; Figure 2a). Results were consistent between sampling years but inconsistent between pollinator species (see Data 4 in Muñoz‐Gallego et al., 2022). *Derelomus chamaeropis* abundance per inflorescence was not impacted by moth herbivory (χ^2^ = 1.52, df = 1, *p* = 0.22; Figure 2b). Conversely, moth‐attacked palms had almost twice as many M. *pallidulus* individuals per inflorescence as unattacked ones (χ^2^ = 4.69, df = 1, *p <* 0.05; Figure 2c). Moreover, moth‐attacked palms showed a 1.4‐fold higher fruit initiation than unattacked ones (χ^2^ = 4.18, df = 1, *p <* 0.05; Figure 2e). Finally, moth herbivory had no effect on fruit development (χ^2^ = 0.15, df = 1, *p =* 0.69; Figure 2f; we used only data from 2020 due to insufficient *N* in 2019). See Data 4 in Muñoz‐Gallego et al. (2022) for further statistical details.

### The isolated effects of goat herbivory on palm reproduction

**FIGURE 3:**
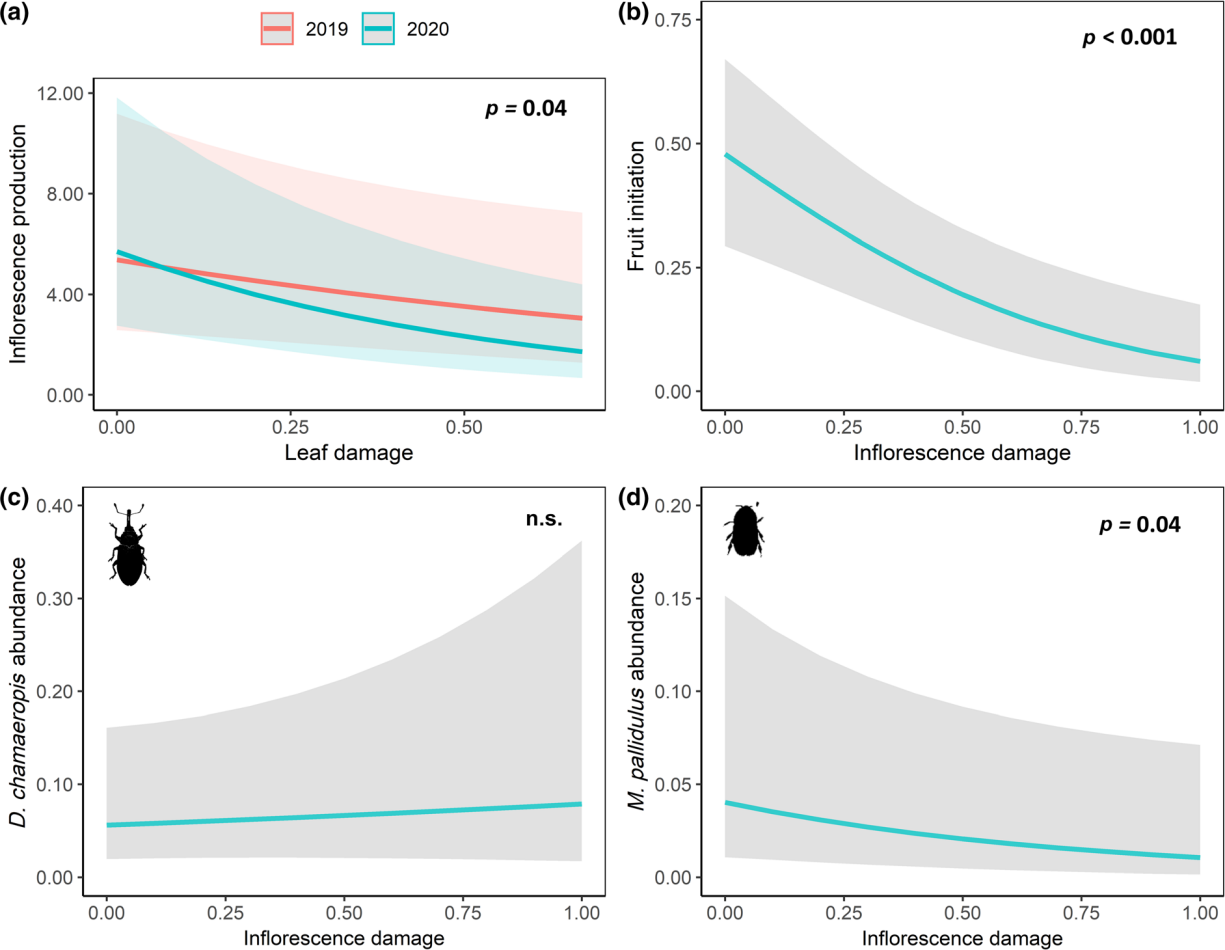
Isolated effects of goat herbivory on palm reproduction. (a) Two‐way interaction between leaf damage and year (2019 and 2020) on inflorescence production (*n* = 69 palms in 2019 and 64 palms in 2020). Regression model predictions about the effect of inflorescence damage on (b) fruit initiation (*n* = 76 palms), (c) *Derelomus chamaeropis* abundance per inflorescence (*n* = 134 palms), and (d) *Meligethinus pallidulus* abundance per inflorescence (*n* = 134 palms). Dark shading indicates 95% confident intervals. The *p* values denote statistically significant effects and n.s. indicates nonsignificant effects.

Feral goats were the main vertebrate consumers of palm inflorescences. During the 10,776 h of recorded camera trapping, 91.4% of the inflorescence damage events (*n* = 58) were performed by feral goats (see Video S1), while only 8.6% by domestic sheep. The proportion of palms attacked (via inflorescence and/or leaf damage) by goats varied among populations and years (see Figure 1d), ranging from 1.8% (PS, 2020) to 92.9% (PF, 2019). From 133 sampled palms, 44.4% showed inflorescence damage (0.47 ± 0.03 mean ± se rate, *n* = 59) and 41.3% experienced leaf damage (0.26 ± 0.02 mean ± se rate, *n* = 55) in March. Inflorescence production was negatively associated with leaf damage (χ^2^ = 8.81, df = 1, *p <* 0.01), although the interaction between leaf damage and year was significant (χ^2^ = 4.26, df = 1, *p <* 0.05; i.e., leaf damage in 2019 and 2020 had different slopes; Figure 3a). Predicted values of the main effect showed that, for example, palms with a leaf damage rate ≥40.0% had between 1.4‐ and 1.8‐fold fewer inflorescences than palms without leaf damage. Moreover, inflorescence damage had a negative effect on the overall pollinator abundance per inflorescence (χ^2^ = 5.27, df = 1, *p <* 0.05, see Appendix S1: Figure S3a). However, separate analyses for each pollinator species revealed a significant and negative effect for *M. pallidulus* (χ^2^ = 4.09, df = 1, *p <* 0.05; Figure 3d) but not effect for *D. chamaeropis* (χ^2^ = 0.04, df = 1, *p* = 0.84; Figure 3c). Palms with an inflorescence damage rate ≥50.0% had between 2.0‐ and 4.0‐fold fewer *M. pallidulus* individuals per inflorescence than undamaged palms. Regarding reproductive success, inflorescence damage as the main factor had a significant negative effect on fruit initiation (χ^2^ = 19.52, df = 1, *p* < 0.001; Figure 3b). For example, palms with an inflorescence damage rate ≥50.0% had a fruit initiation success between 2.4‐ and 8‐fold lower than undamaged palms. Leaf damage showed temporal inconsistency (χ^2^ = 6.54, df = 1, *p <* 0.05; see variation in the slopes between years in Appendix S1: Figure S3b). Conversely, neither inflorescence nor leaf damage affected fruit development (*p =* 0.97 and 0.87, respectively). See Appendix S2: Figure S1 for raw data graphs and Data 4 (Muñoz‐Gallego et al., 2022) for further statistical details.

### The combined effect of both herbivores on palm reproduction

**FIGURE 4:**
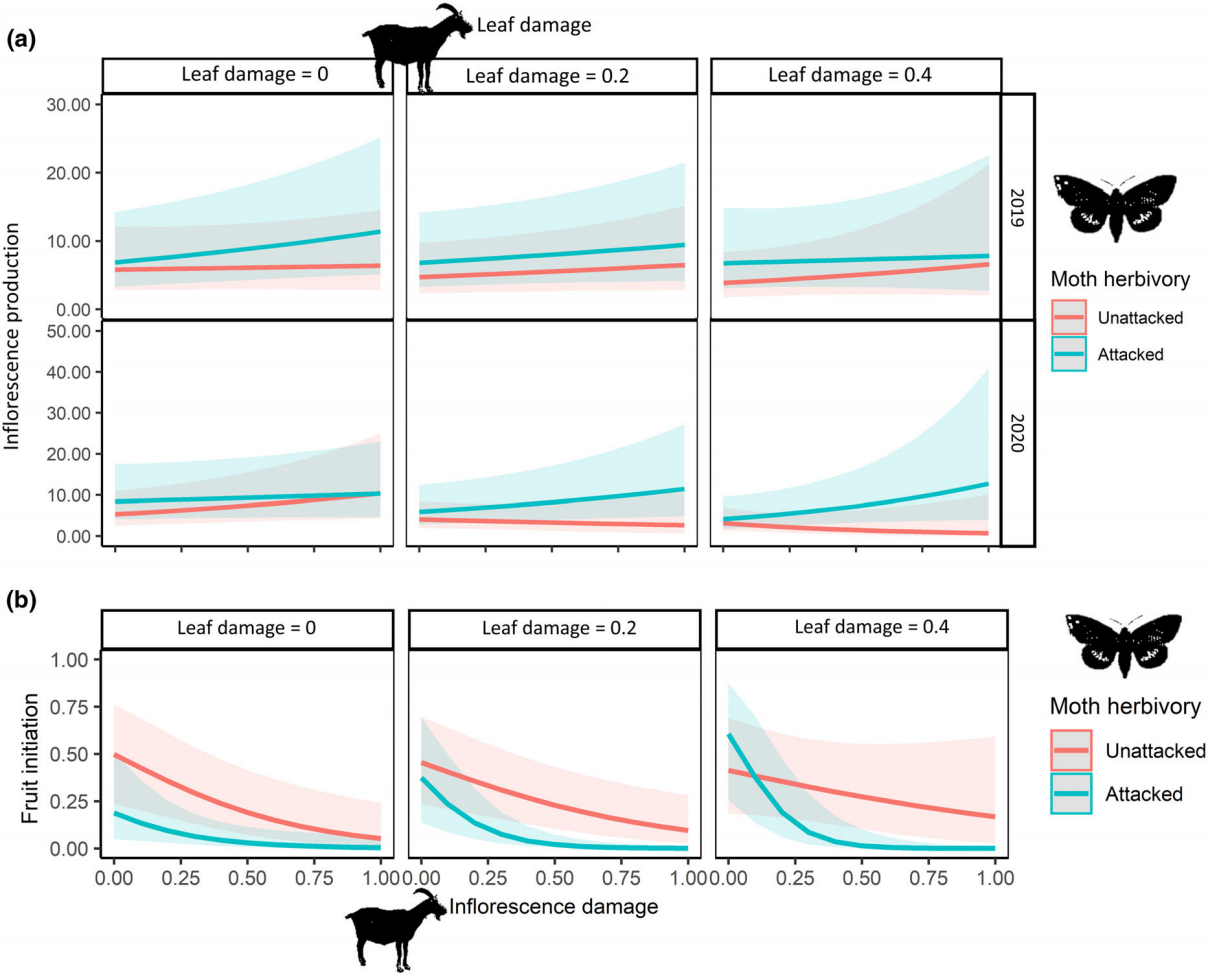
Nonadditive effects of moth herbivory and goat herbivory on palm reproduction. (a) Four‐way interaction among inflorescence damage, leaf damage, moth herbivory, and year (2019 and 2020) on inflorescence production (*n* = 69 unattacked palms and 63 moth‐attacked palms in 2019; 64 unattacked palms and 66 moth‐attacked palms in 2020). (b) Three‐way interaction among inflorescence damage, leaf damage, and moth herbivory on fruit initiation (*n* = 106 unattacked palms and 100 moth‐attacked palms). Dark shading indicates 95% confident intervals.

The three types of herbivory (moth herbivory, inflorescence damage by goat, and leaf damage by goat) had a significant third‐order interaction on inflorescence production, but only in 2020 (χ^2^ = 5.42, df = 1, *p <* 0.05; Figure 4a). Palms, moth‐attacked or not, showed similar inflorescence production at different rates of inflorescence damage when leaf damage was null. However, for high values of leaf and inflorescence damage (say, ≥40.0%), moth‐attacked palms had between 3.8 and 18.4‐fold more inflorescences than unattacked ones. Similar to the isolated effect of goat herbivory (see *The isolated effects of goat herbivory on palm reproduction*), inflorescence damage by goat had a negative effect on the overall pollinator abundance per inflorescence (χ^2^ = 4.37, df = 1, *p <* 0.05; Appendix S1: Figure S4a), being significant only for *M. pallidulus* (χ^2^ = 5.58, df = 1, *p <* 0.05) but not for *D. chamaeropis* (χ^2^ = 0.20, df = 1, *p =* 0.65). Moreover, moth herbivory and leaf damage by goat had an interaction effect on *M. pallidulus* abundance but only in 2019 (χ^2^ = 5.99, df = 1, *p <* 0.05; Appendix S1: Figure S4b). Moth herbivory, inflorescence damage by goat, and leaf damage by goat had a significant interaction effect on fruit initiation (χ^2^ = 3.93, df = 1, *p <* 0.05; Figure 4b). This third‐order interaction indicates inconsistent effect of one herbivory type across levels of other herbivory types. For instance, for null and moderate levels of leaf damage (0–0.2), moth attacked palms had always (i.e., for all levels of inflorescence damage) lower fruit initiation than unattacked palms (Figure 4b). However, for severe leaf damage (≥0.4), moth attacked palms showed higher or lower fruit initiation than unattacked palms depending on the intensity of inflorescence damage (Figure 4b). On the contrary, neither moth herbivory nor goat herbivory as main factors had effect on fruit development (*p* = 0.089). See Appendix S2: Figures S2 and S3 for raw data graphs and Data 4 on Figshare (Muñoz‐Gallego et al., 2022) for further statistical details.

## DISCUSSION

Despite the increasing number of studies about the effects of herbivory on plant performance, the impacts mediated by pollination have still received little attention, particularly on plants with specialized pollination (Haas & Lortie, 2020). Our study contributes to filling this knowledge gap by showing how disparate introduced herbivores (insects and mammals) modulate specialized plant–pollinator interactions and alter plant reproductive success. Thus, we demonstrate that the effects of herbivory can vary widely in strength and sign, depending not only on the herbivore species but also on the pollinator species.

### The effects of herbivory by the invasive moth *P. archon*: Vigor and/or overcompensation?

Although herbivory is typically categorized as a negative interaction, the net outcome on pollination could be neutral or even positive (Haas & Lortie, 2020). Indeed, contrary to our expectations, moth‐attacked palms attracted more pollinators (specifically, *M. pallidulus*), translating into a higher fruit initiation. Two non‐exclusive hypotheses could explain these results: (A) The *plant vigor hypothesis* (Price, 1991), which postulates that herbivores select the most vigorous plants. Plant traits, such as the number of flowers/inflorescences, can simultaneously attract both mutualistic (pollinators) and antagonistic (herbivores) species (Bronstein et al., 2007). In fact, Ruiz et al. (2017) found that *P. archon* prefers larger palms. (B) The *overcompensation hypothesis* (Owen & Wiegert, 1976), by which plants overproduce resources allocated to reproduction to compensate for herbivory (e.g., Aguirrebengoa et al., 2021; Garcia & Eubanks, 2019). Controlled experiments, adding moth larvae to intact palms to remove host selection by the moth, should be carried out to evaluate the suggested hypotheses. Despite these apparently positive effects of moth herbivory on palm reproduction, it is important to point out that stems with a high level of moth herbivory (abundant sawdust and twisted axis) seem to interrupt inflorescence production (R. Muñoz‐Gallego, personal observation), and many of them end up dying (0.33 ± 0.07 mean ± se dead stems per moth‐attacked palm, *n* = 128). Therefore, the net effect of moth herbivory may turn negative as the attack progresses. In addition, other effects of herbivory not investigated here, such as changes in quality of fruits and seeds (Peschiutta et al., 2020) and changes in flowering phenology (Russell‐Mercier & Sargent, 2015), should be considered in future studies in this system.

### Goat herbivory negatively impacted palm reproduction

Inflorescence damage led to significant direct and indirect effects on pollinators and palm reproduction. First, *M. pallidulus* abundance but, interestingly, not *D. chamaeropis* abundance, was strongly reduced when palm suffered high inflorescence damage. The nitidulid *M. pallidulus* seems, thus, to be more sensitive to herbivory, probably because of its feeding habits as “surface grazers” and its life cycle (Jelínek et al., 2011), in which the larval development is more exposed to predators and environmental disturbances. Conversely, the close coevolution between the dwarf palm and its obligated mutualist pollinator *D. chamaeropis* (Anstett, 1999) in environments with many herbivores and other disturbances (fire, e.g., Jácome‐Flores et al., 2018) might ensure the successful pollinator' reproduction, preventing pollination failure (Wilcock & Neiland, 2002). Secondly, fruit initiation was negatively affected by inflorescence damage, reducing the final crop size significantly. In Mallorca, many dwarf palm populations suffer critical rates of inflorescence damage. In the PF population, around 80%–90% of the palms (*n* = 212) had no inflorescences at the end of the springs of 2019 and 2020 (Muñoz‐Gallego et al., 2022). Consequently, a significant long‐term impact on the palm demography could arise (Flaherty et al., 2018). Finally, the negative relationship between inflorescence production and leaf damage could be explained by a reallocation of resources to defense mechanisms (Lucas‐Barbosa, 2016), or simply because of resource use (i.e., goats prefer to feed on inflorescences but they shift to feed on leaves when the former become scarce). Considering the noted reproductive role of the dwarf palm's leaves in attracting pollinators (Dufaÿ et al., 2003), investigating whether leaf damage alters VOCs' emission and, thus, affects palm pollination and reproductive success represents an intriguing pending task.

### Nonadditive effects of both herbivores on palm reproduction

Assessing interaction effects can be crucial to uncovering unpredictable impacts on biological systems (Côté et al., 2016), like those reported on plant reproduction (Aguirrebengoa et al., 2021; Gagic et al., 2016). Interestingly, in our study system, both moths and goats exerted some unexpected nonadditive effects on inflorescence production and fruit initiation, since they differed from the isolated effects of each herbivore. For example, inflorescence production was not impacted by the isolated effect of moth herbivory but was negatively affected by goat herbivory (leaf damage). Conversely, moth‐attacked palms simultaneously suffering high rates of inflorescence and leaf damage by goat had more inflorescences than unattacked palms (although only in 2020). Although the interaction between moth and goat herbivories had an apparent positive effect on inflorescence production, the final crop size was drastically reduced mainly by two mechanisms: (1) the intense consumption of inflorescences by goats, especially in early spring (March), and (2) the fruit initiation decrease when palms were moth‐attacked and, simultaneously, affected by high rates of flower damage and leaf damage by goats. The simultaneous incomes of three different types of damage (stem, leaves, and inflorescences) could demand many plant resources, decreasing those to reproduction (Bronstein et al., 2007; Stephens et al., 2013). Therefore, the net combined effect of both herbivores on the dwarf palm reproduction was negative.

## CONCLUSIONS

Our findings support that the combined effect of contrasting herbivores, such as one invertebrate and one vertebrate, on plant reproductive success can result in highly diverse and unexpected outcomes (Stephens et al., 2013). The herbivory effects on the reproduction of the pollination‐specialized dwarf palm differed between herbivore species (positive effects by *P. archon* versus negative effects by *C. hircus*) but also between pollinator species (neutral effects on *D. chamaeropis* versus positive or negative effects on *M. pallidulus*). It is well known that the Mediterranean dwarf palm is a long‐lived plant, highly tolerant of environmental and anthropogenic disturbances (Benarchid et al., 2020; García et al., 2018; Jácome‐Flores et al., 2018). However, it is unquestionable that the most sensitive palm populations, like the insular ones, could be seriously impacted in the long term by the combined effects of these introduced and highly abundant herbivores. We call for future long‐term studies that investigate the joint impact of introduced herbivores on plant species, especially rare or specialized ones for which appropriate management plans are most required for their conservation.

## CONFLICT OF INTEREST

The authors declare no conflict of interest.

## Supporting information


Appendix S1

Appendix S2

Video S1

Video S1 Legend

## Data Availability

Data and statistics (Muñoz‐Gallego et al., 2022) are available on Figshare at https://doi.org/10.6084/m9.figshare.19780288.v4.
