# Supplementary material for: Nonadditive effects of two contrasting introduced herbivores on the reproduction of a pollination‐specialized palm
Source: Ecology. 2022 Jul 27;103(11):e3797. doi: 10.1002/ecy.3797 (PMC9787982; doi:10.1002/ecy.3797)
Supplement: Supplementary file 1 — Appendix S1 [file ECY-103-e3797-s001.pdf]

Appendix S1

Article title: Non-additive effects of two contrasting introduced herbivores on the reproduction of a pollination-specialized palm

Journal: Ecology

Authors: Raquel Muñoz-Gallego[1]*, Jose M. Fedriani[2,3], Pau E. Serra[1] & Anna Traveset[1]

[1] Global Change Research Group, Mediterranean Institute of Advanced Studies (IMEDEA, CSIC-UIB), (C/ Miquel Marquès, 21, 07190, Esporles, Balearic Islands, Spain)

[2] Desertification Research Centre (CIDE, CSIC), (Crta. Moncada-Náquera, Km 4.5, 46113, Moncada, Valencia, Spain)

[3] Doñana Biological Station (EBD, CSIC), (C/Americo Vespucio s/n, 41092 Seville, Spain).

*Corresponding author: Raquel Muñoz Gallego, rmunoz@imedea.uib-csic.es

Figure S1. Palm study populations in Mallorca (Balearic Islands, Spain): *EB*, Ermita de Betlem; *PS*, Puig de Santuïri; *PF*, Platja de Formentor; *CV*, Campament de la Victòria.

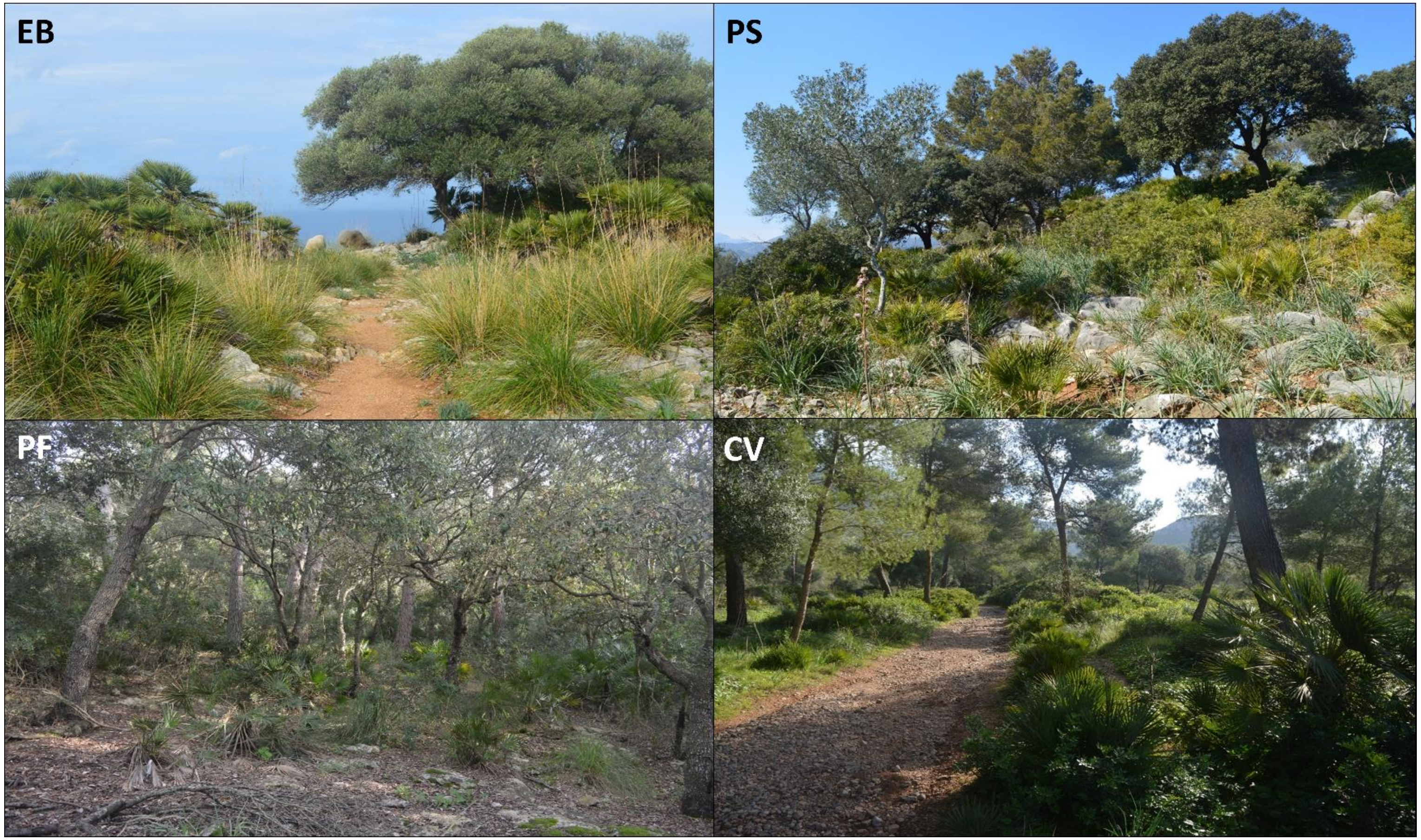

Figure S2. The fate of female inflorescences was sampled to estimate the effect of (a) moth herbivory and (b) goat herbivory on fruit initiation and fruit development for two sampling years (2019 and 2020). Fruit initiation was estimated in June and fruit development in September. Inflorescence fate is indicated in different colors: “Set fruit” = inflorescences that continued their development, “Aborted” = inflorescences that stopped their development, “Consumed” = inflorescences partially or fully consumed by goat, “NA” = missing data. Sample size, i.e. number of female inflorescences, is shown within each color bar.

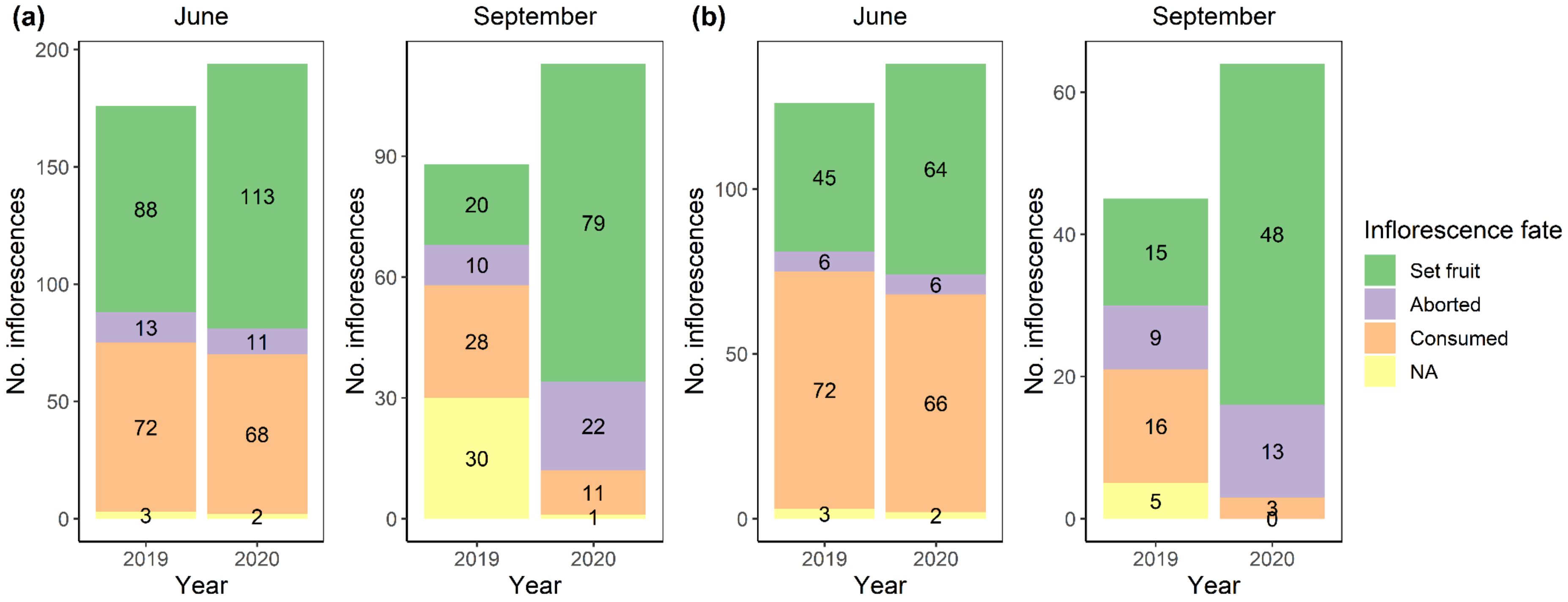

Figure S3. Isolated effects of goat herbivory on palm reproduction. (a) Regression model predictions about the effect of inflorescence damage on the overall pollinator abundance per inflorescence (n = 134 palms). (b) Two-way interaction plot between leaf damage and sampling year (2019 and 2020) on fruit initiation (n = 37 palms in 2019 and 39 palms in 2020). Dark shading indicates 95 % confident intervals. *P* values denote statistically significant effects.

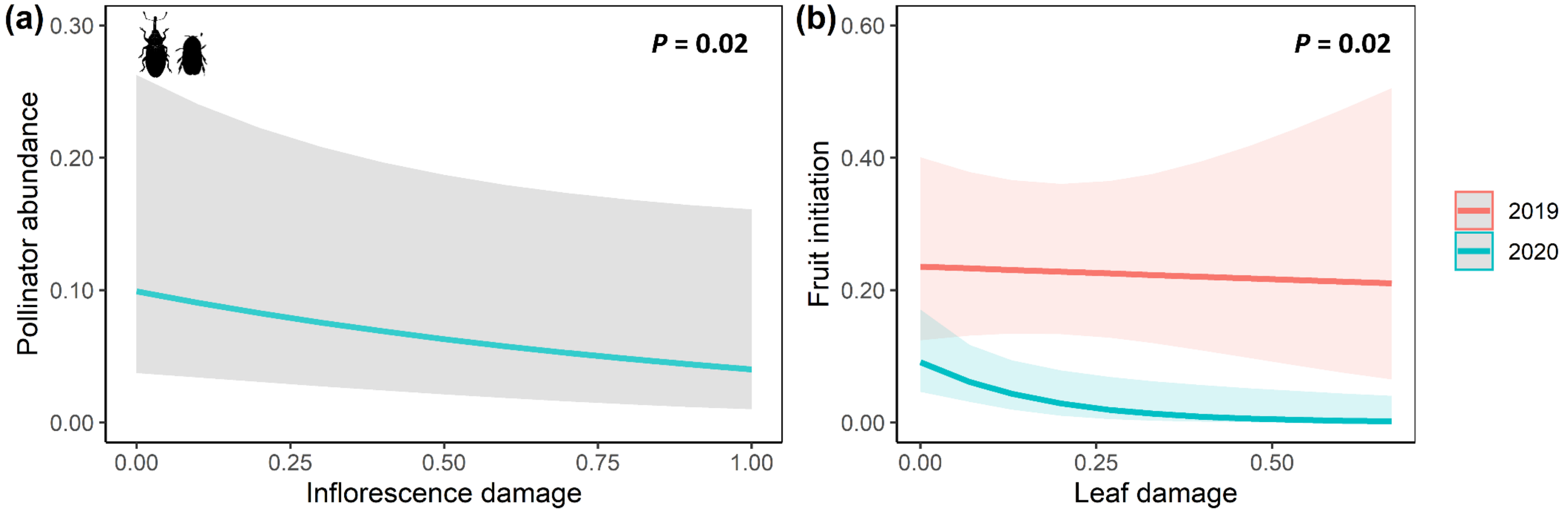

Figure S4. Interaction effects of moth herbivory and goat herbivory on palm reproduction. (a) Regression model predictions about the effect of inflorescence damage on the overall pollinator abundance per inflorescence (n = 262 palms). *P* values denote statistically significant effects. (b) Three-way interaction among moth herbivory, leaf damage, and sampling year (2019 and 2020) on *M. pallidulus* abundance per inflorescence (n = 69 unattacked and 63 moth-attacked palms in 2019, 64 not moth-attacked and 66 moth-attacked palms in 2020). Dark shading indicates 95 % confident intervals.

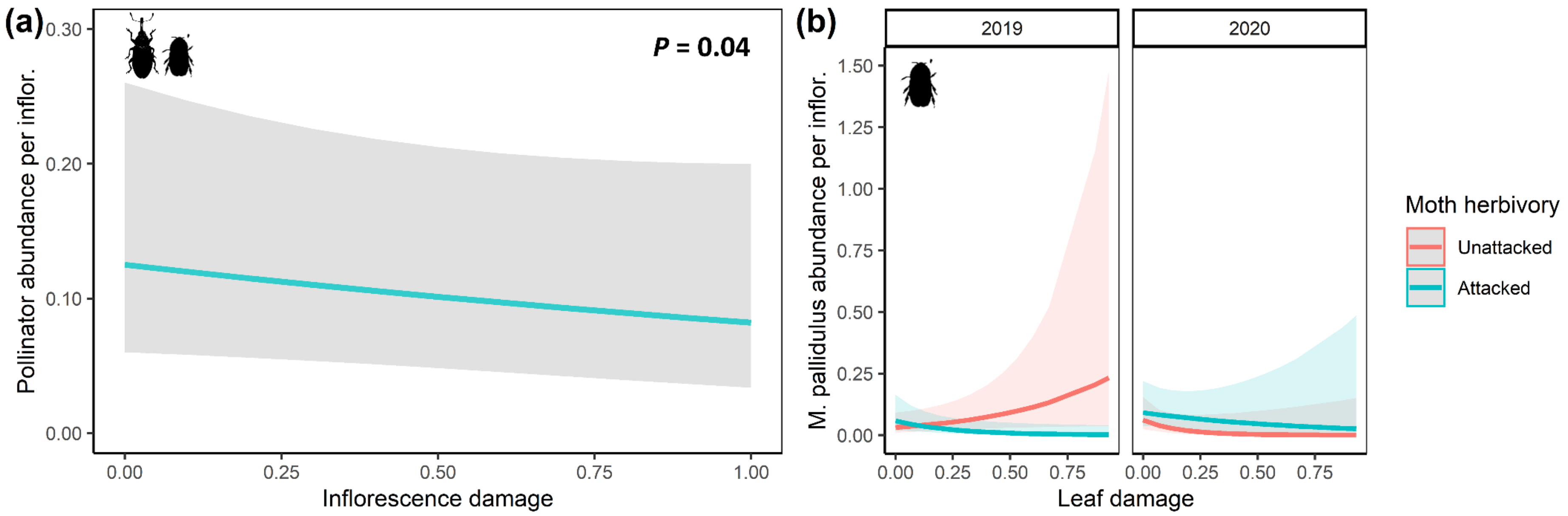

Table S1. Other inflorescence visitors observed in the dwarf palms during the visual censuses carried out in Mallorca during the springs of 2019 and 2020. Frequency of occurrence was calculated as the number of inflorescences where each visitor was observed divided by the total number of inflorescences (n = 899) sampled in both years (2019 and 2020) multiplied by 100. Those that could not be registered are indicated as *NA*.

**Phylum**	**Class**	**Order**	**Family**	**Species**	**Frequency of occurrence (%)**
Arthropoda	Arachnida	Araneae	-	-	1.00
	Diplopoda	Julida	-	-	0.78
	Diptera	-	-	-	*NA*
	Insecta	Blattodea	-	-	0.67
		Coleoptera	Tenebrionidae	*Isomira murina*	1.33
				Lagria hirta	0.33
		Dermaptera	Forficulidae	*Forficula auricularia*	0.44
		Hymenoptera	Apidae	*Apis mellifera*	0.11
			Formicidae	-	30.92
		Thysanoptera	-	-	*NA*
	Malacostraca	Isopoda	-	-	4.45
Mollusca	Gastropoda	Pulmonata	-	-	*NA*

Camera trapping methodology. Camera-traps (LTL ACORN 5310A, detection range = 18 m) were set in front of palms, from half to five meters' distance, over 24-hours a day for 14 consecutive days on average (336.75 ± 21.46 hours per palm). Cameras were set up to record a 10 seconds-video automatically when any movement occurred, with 30 seconds of delay between each capture to prevent memory cards from filling up. We considered as independent inflorescence damage events those separated by more than 5 minutes. The recording was finished when no more inflorescences were left or these had dried up. Thus, for each palm the rate of inflorescence damage events was estimated as the total number of inflorescence damage events divided by the number of camera active days.
