# Supplementary material for: Nonadditive effects of two contrasting introduced herbivores on the reproduction of a pollination‐specialized palm
Source: Ecology. 2022 Jul 27;103(11):e3797. doi: 10.1002/ecy.3797 (PMC9787982; doi:10.1002/ecy.3797)

Appendix S2

Article title: Non-additive effects of two contrasting introduced herbivores on the reproduction of a pollination-specialized palm

Journal: Ecology

Authors: Raquel Muñoz-Gallego[1]*, Jose M. Fedriani[2,3], Pau E. Serra[1] & Anna Traveset[1]

[1] Global Change Research Group, Mediterranean Institute of Advanced Studies (IMEDEA, CSIC-UIB), (C/ Miquel Marquès, 21, 07190, Esporles, Balearic Islands, Spain)

[2] Desertification Research Centre (CIDE, CSIC), (Crta. Moncada-Náquera, Km 4.5, 46113, Moncada, Valencia, Spain)

[3] Doñana Biological Station (EBD, CSIC), (C/Americo Vespucio s/n, 41092 Seville, Spain).

*Corresponding author: Raquel Muñoz Gallego, rmunoz@imedea.uib-csic.es

Figure S1. Figure 3 from the main document including raw data points.

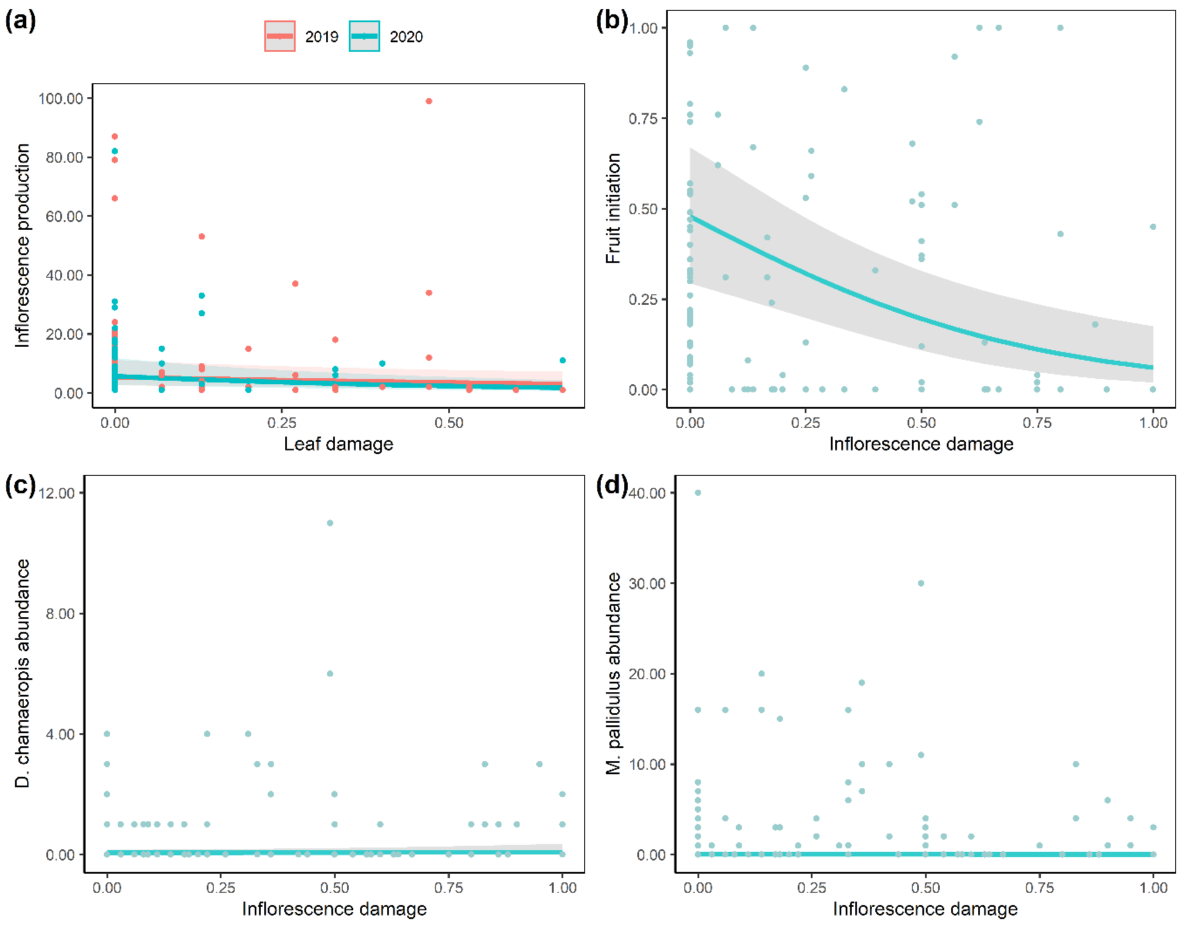

Figure S2. Figure 4a from the main document including raw data points for (a) 2019 and (b) 2020.

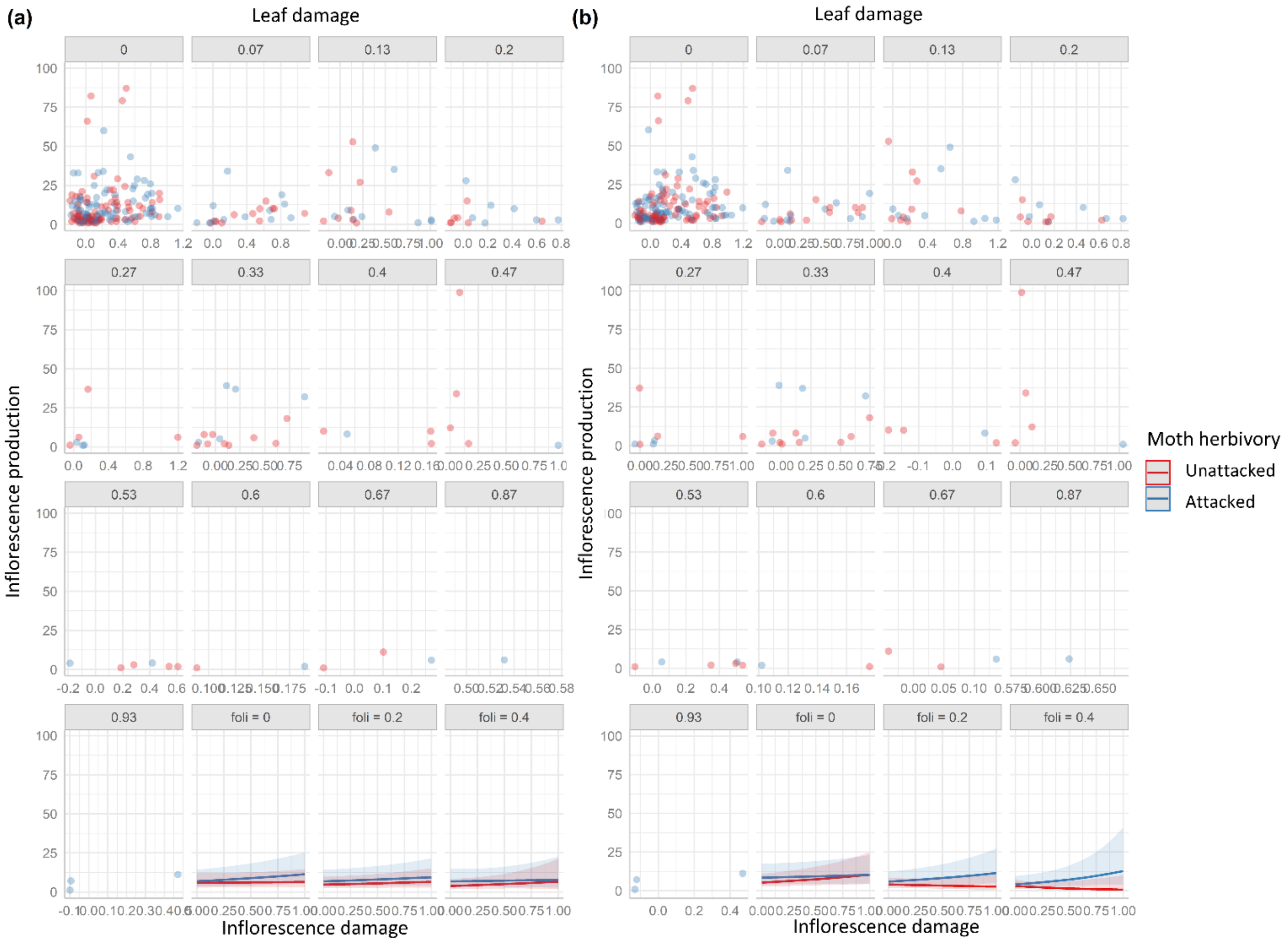

Figure S3. Figure 4b from the main document including raw data points.

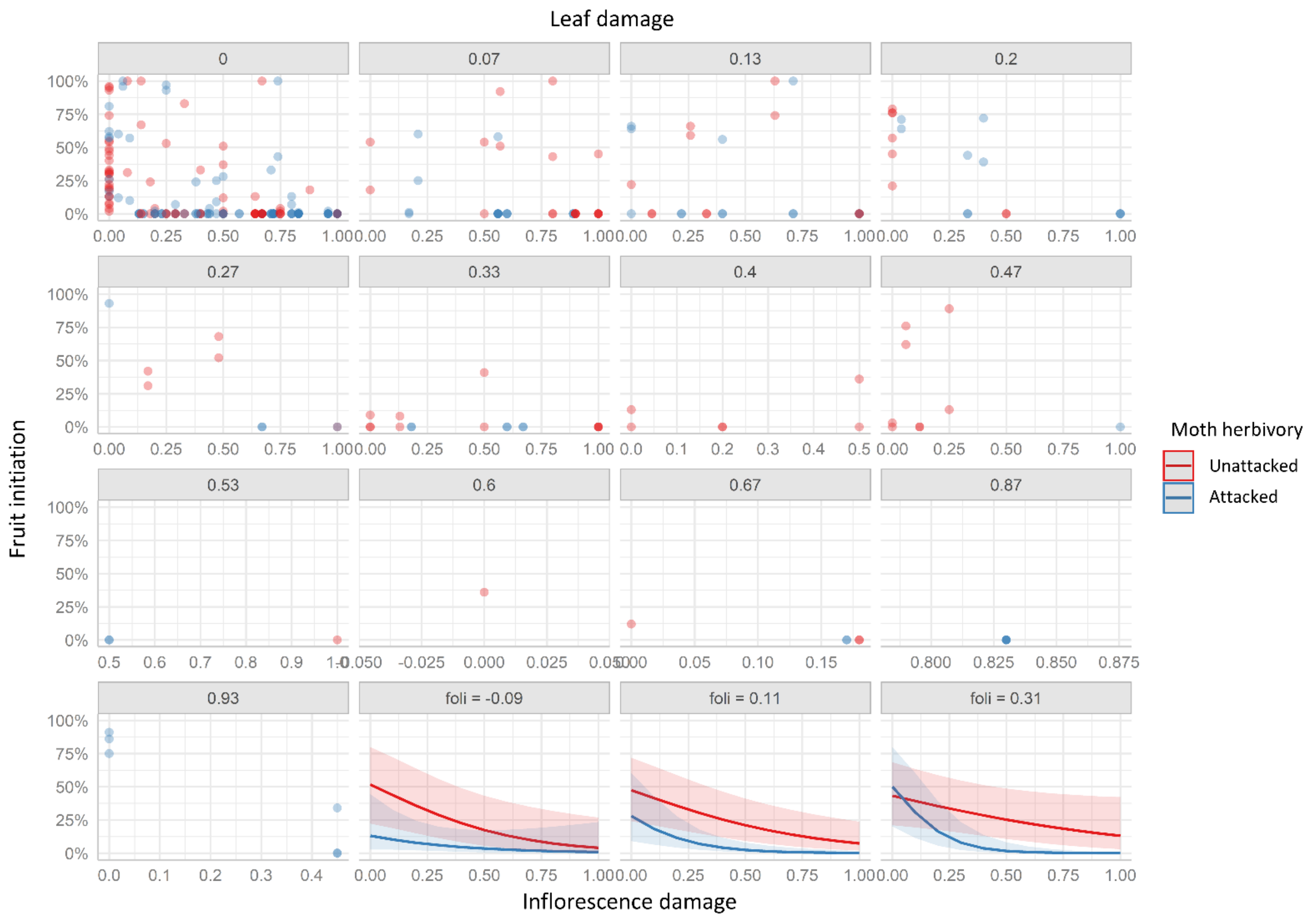

Supplement: Supplementary file 2 — Appendix S2 [file ECY-103-e3797-s004.pdf]
