# Supplementary material for: Nonadditive effects of two contrasting introduced herbivores on the reproduction of a pollination‐specialized palm
Source: Ecology. 2022 Jul 27;103(11):e3797. doi: 10.1002/ecy.3797 (PMC9787982; doi:10.1002/ecy.3797)
Supplement: Supplementary file 4 — Video S1 Legend [file ECY-103-e3797-s003.pdf]

Article title: Non-additive effects of two contrasting introduced herbivores on the reproduction of a pollination-specialized palm

Journal: Ecology

Authors: Raquel Muñoz-Gallego[1]*, Jose M. Fedriani[2,3], Pau E. Serra[1] & Anna Traveset[1]

[1] Global Change Research Group, Mediterranean Institute of Advanced Studies (IMEDEA, CSIC-UIB), (C/ Miquel Marquès, 21, 07190, Esporles, Balearic Islands, Spain)

[2] Desertification Research Centre (CIDE, CSIC), (Crta. Moncada-Náquera, Km 4.5, 46113, Moncada, Valencia, Spain)

[3] Doñana Biological Station (EBD, CSIC), (C/Americo Vespucio s/n, 41092 Seville, Spain).

*Corresponding author: Raquel Muñoz Gallego, rmunoz@imedea.uib-csic.es

Video S1 Legend

Feral goat eating *Chamaerops humilis* inflorescences recorded by camera-trapping (camera-trap LTL ACORN 5310A, detection range = 18 m) on 04/27/2020 in Mallorca (Balearic Islands, Spain). Note that it is a composition of video fragments at x1.5 speed.
